# Design, Synthesis, Molecular Modeling and Biological Evaluation of Novel Pyrazole Benzimidazolone Derivatives as Potent Antioxidants

**DOI:** 10.3390/ph16121648

**Published:** 2023-11-24

**Authors:** Mohamed Adardour, Marouane Ait Lahcen, Mehdi Oubahmane, Walid Ettahiri, Ismail Hdoufane, Hafida Bouamama, Mohammed M. Alanazi, Driss Cherqaoui, Mustapha Taleb, Elena Zaballos Garcia, Abdesselam Baouid

**Affiliations:** 1 Molecular Chemistry Laboratory, Department of Chemistry, Semlalia Faculty of Sciences, 2390, Cadi Ayyad University, Marrakech 40001, Morocco; m.aitlahcen.ced@uca.ac.ma (M.A.L.); mehdi.oubahmane@ced.uca.ma (M.O.); walid.ettahiri@usmba.ac.ma (W.E.); i.hdoufane@uca.ac.ma (I.H.); cherqaoui@uca.ac.ma (D.C.); baouid@uca.ac.ma (A.B.); 2 Laboratory of Engineering, Electrochemistry, Modeling and Environment, Faculty of Sciences, Sidi Mohamed Ben Abdellah University, Fez 30000, Morocco; mustaphataleb62@yahoo.fr; 3 Laboratory of Sustainable Development and Health Research, Faculty of Sciences and Techniques, Cadi Ayyad University, Marrakech 40000, Morocco; h.bouamama@uca.ac.ma; 4 Department of Pharmaceutical Chemistry, College of Pharmacy, King Saud University, P.O. Box 2457, Riyadh 11451, Saudi Arabia; mmalanazi@ksu.edu.sa; 5 Sustainable Materials Research Center (SUSMAT-RC), University of Mohammed VI Polytechnic, Benguerir 43150, Morocco; 6 Department of Organic Chemistry, Faculty of Pharmacy, University of Valencia, Ave. Vte. Andres Estelles s/n, 46100 Valencia, Spain; elena.zaballos@uv.es

**Keywords:** pyrazolyl-benzimidazolone, antioxidant activity, FRAP, TAC, molecular docking

## Abstract

In the present study, we used benzimidazolone as a starting material to efficiently synthesize several hybrid compounds of pyrazole benzimidazolone derivatives by the 1,3-dipolar cycloaddition reaction. These compounds were obtained in average yields and were characterized by NMR (^1^H and ^13^C) and HRMS analysis. The antioxidant activity of the synthesized compounds **5**(**a**–**c**) and **6**(**a**–**c**) was evaluated using in vitro reduction assays, including ferric reducing antioxidant power (FRAP) and total antioxidant capacity (TAC). The results indicated that products **5c**, **6b**, and **6c** exhibit higher antioxidant activity compared to the reference compounds and showed a remarkable ability to effectively remove the radical at IC_50_ (14.00 ± 0.14, 12.47± 0.02, and 12.82 ± 0.10 µM, respectively) under the TAC assessment. Conversely, compound **6c** showed excellent activity at IC_50_ (68.97 ± 0.26 µM) in the FRAP assay. We carried out molecular docking and dynamics simulations to investigate the binding mode and stability of **5c**, **6b**, and **6c** in the active site of human Peroxiredoxin 5. An ADMET study was conducted to determine the drug properties of the synthesized compounds.

## 1. Introduction

Oxidative stress is a primary cause of illnesses such as cancer, aging, atherosclerosis and rheumatoid arthritis, cardiovascular, autoimmune, and neurological disorders. It is produced by the existence of reactive oxygen and nitrogen species (ROS and RNS) in the body, such as peroxides (H_2_O_2_) and free radicals. Antioxidants play an essential role in the body’s defense mechanism by eliminating or regulating the generation and elimination of ROS and RNS. A good balance between ROS, RNS, and antioxidants is essential for proper physiological functioning [1].

Therefore, the design and synthesis of novel molecules capable of serving as potent antioxidants with high effectiveness and low toxicity is a burgeoning topic of research in medicinal chemistry. Heterocyclic compounds containing heteroatoms such as nitrogen, sulfur, and oxygen represent an important class of organic compounds and antioxidant agents with potent free radical scavenging powers [2,3,4]. Pyrazoles continue to be favored as the preferred scaffold, given that compounds in this class showed to be effective in quenching free radicals. Therefore, they hold great promise for further exploration as lead candidates in drug discovery efforts aimed at combating oxidative damage [5,6].

The benzimidazolone derivatives are essential structures in numerous synthetic compounds and have applications in medicinal chemistry. They are useful in the development of new molecules of pharmaceutical or biological interest [7,8,9]. These compounds are known for their antiviral [10,11], anti-inflammatory [12,13,14], anticancer [15,16,17], antimicrobial [18,19,20], anti-HIV [21], anti-diabetes [22,23], and anticonvulsant [24] activities. Furthermore, benzimidazole compounds possess biological properties, including antioxidant activities [25,26,27].

Finding potent heterocyclic molecules containing nitrogen with efficient biological activities is a current strategy. An example of this strategy is the biological applications of pyrazole derivatives as potent acetylcholinesterase inhibitors [28]. Another example is the use of pyrano[2,3-c]pyrazole and pyrazolo[4′,3′:5,6]pyrano[2,3-d]pyrimidine derivatives for their antimicrobial, antioxidant, and anticancer activities [29]. In addition, the 1-methyl-3-propyl-1*H*-pyrazole derivatives have shown their potential as anti-tubercular agents, as evidenced by their in vitro assessment [30]. Pyrazole is an active scaffold and a versatile lead molecule in pharmaceutical development and has interesting biological activity. They are active molecules in medicinal chemistry because they are easy to synthesize, and their chemical structures can be modified at multiple sites. These compounds exhibit a variety of cytoprotective and modulatory functions, which lead to several therapeutic activities such as anticancer effects [31,32], antimicrobial properties [33,34,35], antifungal agents [36,37], and various other functions [38,39,40].

**Figure 1:**
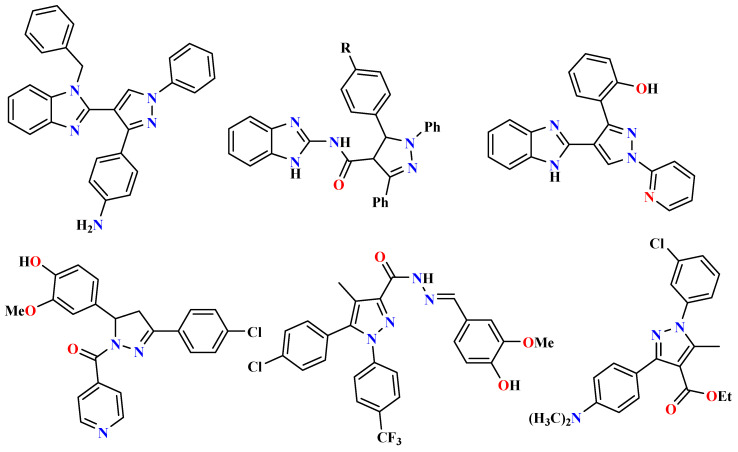
Selected structures of compounds containing pyrazole as antioxidants.

On the other hand, several studies indicated that pyrazole ring systems play a special role in the preparation of new active biological agents with remarkable antimicrobial [41,42], anti-ulcer [43] and antitumor [32,44] activities. The pyrazoles with a benzimidazole exhibit show various biological and pharmacological activities, including anti-inflammatory [45] and anti-ulcerogenic [43]. Pyrazole derivatives are synthesized using the the1,3-dipolar cycloaddition reaction [46,47] and other methods [48,49,50]. Because of this, various pyrazole-benzimidazole hybrids have gained a reputation as potent antioxidants due to their remarkable effectiveness in combating oxidative stress [51,52,53]. Several studies have focused on the design and synthesis of compounds containing pyrazole moiety as antioxidant, as shown in Figure 1. Thus, it was interesting to investigate the synthesis of pyrazolyl-benzimidazolones by 1,3-dipolar cycloaddition and to characterize and evaluate their biological activities. It can be considered a fundamental structural element in exploring and advancing novel drug prospects that offer improved efficacy and reduced toxicity.

**Figure 2:**
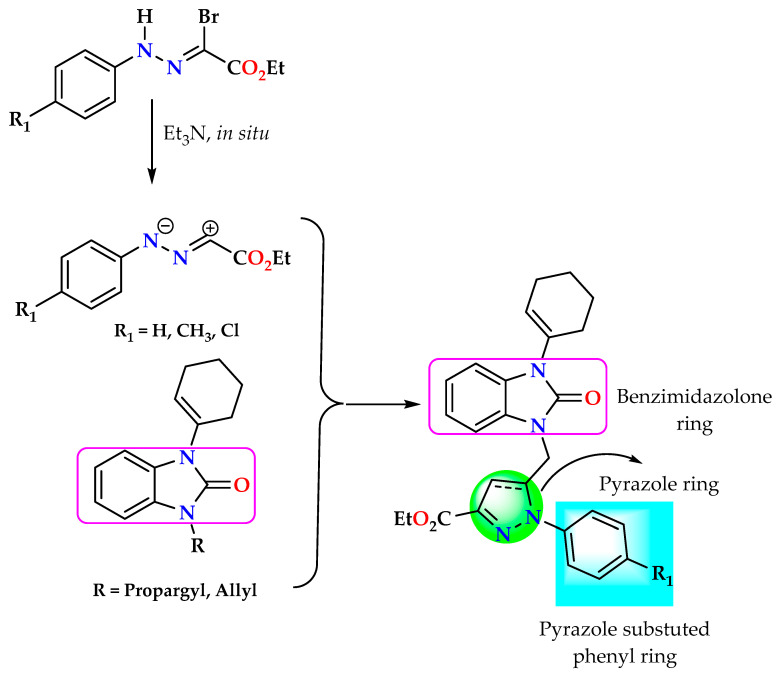
General structure of synthesized pyrazolyl-benzimidazolones.

Therefore, this study aimed to develop the synthesis of new heterocyclic systems containing benzimidazolone and the pyrazole motif (Figure 2). Thus, we synthesized the compound hybrids by the 1,3-dipolar cycloaddition reaction between *N*-alkylated benzimidazolones and nitrilimine derivatives. The antioxidant activity of the synthesized pyrazoles was investigated by assessing their ability to inhibit scavenger radicals. This evaluation used the total antioxidant capacity (TAC) and ferric reducing antioxidant power (FRAP) methods. Finally, the pharmacokinetic and toxicological properties of the synthesized compounds were predicted and investigated. Molecular docking and molecular dynamic simulations were conducted for the most potent derivatives within the active site of human peroxiredoxin 5 (PRDX5) to investigate their potential binding conformation and stability.

## 2. Results and Discussion

### 2.1. Chemistry

**Scheme 1:**
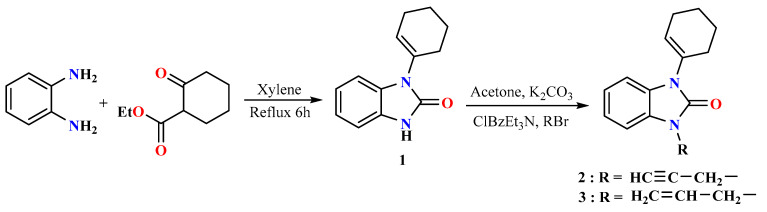
Synthetic route of the compounds **2** and **3**.

**Scheme 2:**
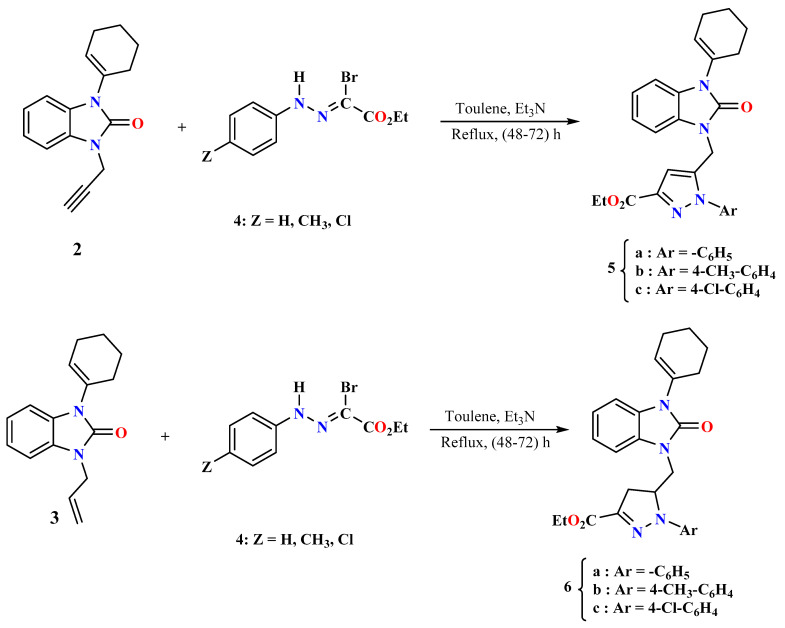
Synthetic route of the heterocyclic compounds **5** and **6**.

The synthesis of novel pyrazole benzimidazolone derivatives consists of three main steps (Scheme 1 and Scheme 2). In the first step, the 1-(cyclohex-1-enyl)-1,3-dihydro-2*H*-benzimidazol-2-one (**1**) was efficiently synthesized by the condensation of keto-ester and o-phenylenediamine in xylene at reflux for 6 h [54]. In the second step, the *N*-alkylation of benzimidazolone with propargyl bromide and allyl bromide was carried out using potassium carbonate and benzyl triethylammonium chloride as a catalyst in acetone at room temperature for 4–6 h, providing the *N*-propargyl-benzimidazolone **2** and *N*-allyl-benzimidazolone **3** (Scheme 1). In the final step, compounds **2** and **3** were reacted with *N*-aryl-C-ethoxycarbonylnitrilimine precursors in toluene at reflux for 48–72 h to give the pyrazolyl-benzimidazolone derivatives **5** and **6**.

Continuing our previous works concerning synthesizing new heterocyclic systems [54,55,56], we are interested in preparing new heterocyclic systems containing benzimidazolone rings. To this end, we have prepared the dipoles and the alkylated benzimidazolones, and the latter have dipolarophilic sites that can react with the synthesized nitrilimines. Next, we examined the condensation of benzimidazolone **2** or **3** with *N*-aryl-C-ethoxycarbonylnitrilimines, generated in situ by triethylamine on ethylhydrazono-α-bromoglyoxylate **4**. Indeed, when heating dry toluene at reflux for 48–72 h, we obtained only one type of cycloadduct **5** and **6** (Scheme 2) with a yield of 60–75% in all studied cases. These products are formed through a monocondensation reaction of the dipole with the triple bond present in the propargyl group or double bond in the allyl group. It should be noted that no product resulting from the addition of the dipole to the intracyclic dipolarrophilic carbon–carbon site of cyclohexenyl was isolated. Similarly, no addition of the dipole to the carbon–oxygen double bond was observed, regardless of the amount of dipole used.

We established the structures of the synthesized heterocycles **5** and **6** based on spectral data (^1^H, ^13^C NMR, and HRMS). The multiple bonds of propargyl-benzimidazolone **2** and allyl-benzimidazolone **3** are the most reactive sites through the dipole. The ^1^H NMR spectra of the isolated products **5**(**a**–**c**) shows three singlets at 6.49, 6.50, and 6.52 ppm assigned to the resonance of the pyrazole proton at the 4’ position. In the ^13^C NMR spectrum of the compounds, **5**(**a**–**c**) present three peaks at 109.50, 109.33, and 109,88 ppm attributed to the carbon in the 4’ position.

In the ^1^H NMR spectra of **6**(**a**–**c**), these compounds display a multiplet at 5.15, 5.31, and 5.01 ppm due to the pyrazolic proton in the 5’ position. On the ^13^C NMR spectrum, we note the appearance of new signals at 58.77, 61.80, and 58.72 ppm, attributable to the methylenic carbons at the 5’ position, respectively, of the pyrazoline ring.

### 2.2. Antioxidant Activity by TAC and FRAP Methods

Antioxidant assays using FRAP and TAC methods were used to assess the antioxidant activities of the synthesized compounds **5**(**a**–**c**) and **6**(**a**–**c**). The results are illustrated in Table 1.

In the series of pyrazoles **5**(**a**–**c**) and **6**(**a**–**c**), all compounds showed the reducing power for molybdenum (VI) in TAC. Products **5** and **6** demonstrated moderate to high antioxidant activity. In particular, the synthesized compounds **5c**, **6b**, and **6c** exhibited excellent antioxidant activity with IC_50_ values of 14.00 ± 0.14, 12.47 ± 0.02, and 12.82 ± 0.10 µM, respectively. These values were significantly better than the reference compounds used in the study (ascorbic acid (88.12 ± 0.23 µM) and BHT (31.76 ± 1.22 µM)).

Another antioxidant property was the comparative capacity of the pyrazole derivatives in terms of the FRAP. In Table 1, we can see that the results of the FRAP assay show that the IC_50_ values are significantly better compared to the commercial reference (ascorbic acid). The **5a**, **6a,** and **6c** are the most active compounds in this method, with IC_50_ values of 108.30 ± 0.59, 92.70 ± 0.43, and 68.97 ± 0.26 µM, respectively. These results are comparable to ascorbic acid’s IC_50_ value (88.12 ± 0.23 µM). The FRAP experiments show that compound **6c** has a significantly higher ferric reduction capacity than compounds **5a** and **6a**.

Due to their significant importance, we also analyzed the structural attributes of the synthetic pyrazoles as antioxidant compounds through the prism of structure–activity relationships (SAR). The presence of methyl or chloro in the phenyl group within the pyrazole ring significantly affected the antioxidant activity. Furthermore, the inclusion of electron-donating substituents such as methyl in compound **6b** (12.47 ± 0.02 µM) and electron-withdrawing substituents like chloro in compounds **5c** (14.00 ± 0.14 µM) and **6c** (12.82 ± 0.10 µM), in the para position of the phenyl group attached to the pyrazole ring, resulted in an increase in the antioxidant activity compared to compounds **5a** (44.62 ± 0.20 µM) and **6a** (56.55 ± 0.10 µM). Moreover, pyrazole derivatives **5b** and **6b** do not have the same effect, and this equipotent activity could be related to the presence of the conjugated pyrazole ring in compound **6b**. Conversely, the analysis of FRAP showed that the synthesized compounds have moderate activity levels compared to ascorbic acid. From the observed values, we can conclude that the pyrazole ring likely plays a significant role in quenching or suppressing the radicals in this assay. Previous reports [52,57,58] indicate that compounds possessing structures with substituted phenyl moieties, especially with functional groups like para-methyl and para-chloro, may exhibit metal chelating activity.

### 2.3. Molecular Docking of the Selected Compounds

**Figure 3:**
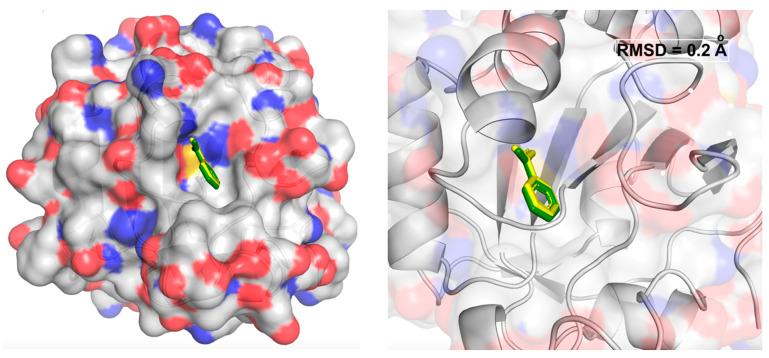
The co-crystallized (green) and re-docked (yellow) benzoic acid in the active site of PRDX5 protein, including the RMSD of atomic positions.

The precision of our docking procedure was confirmed by the redocking of benzoic acid into the active site of the enzyme, resulting in an RMSD value of 0.20 Å (Figure 3). This RMSD value is less than 2 Å, indicating the reliability of the docking protocol [59]. The docking results were analyzed using the Discovery Studio Visualizer and Pymol software (The PyMOL Molecular Graphics System; Version 1.8; Schrödinger LLC: New York, NY, USA, 2015).

Following the in vitro findings, we conducted some profound docking studies to investigate the possible binding modes of the most active compounds **5c**, **6b**, and **6c**, alongside reference compounds (ascorbic acid, BHT, and benzoic acid) within the active site of human peroxiredoxin 5 (PRDX5 (PDB ID: 1HD2). The binding affinity values are presented in Table 2, along with the interaction details. We found that the binding affinity values of the synthesized molecules (≤−6.0 Kcal/mol) were higher compared to the reference compounds (−4.6 Kcal/mol) within the active site of 1HD2.

**Figure 4:**
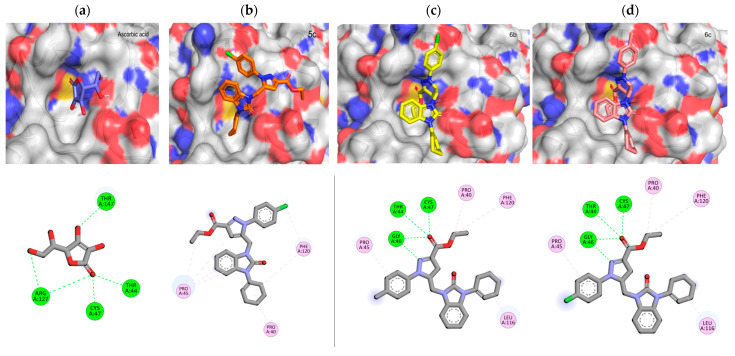

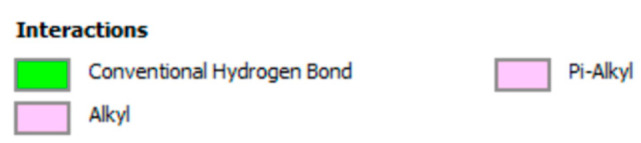
The 3D and 2D representation of molecular docked complex of ascorbic acid (**a**), **5c** (**b**), **6b** (**c**), and **6c** (**d**) with the active site of PRDX5 (PDB ID: 1HD2).

Considering the information on the human peroxiredoxin family and the specific role of Cys47, a conserved residue at the N-terminus of the bent helix 2, shared by all peroxiredoxins, has been attributed to peroxide catalysis. In addition, the active site of 1HD2 comprises conserved residues Thr44, Gly46, Thr147, Pro40, Pro45, Phe120, Arg127, and Leu149, all involved in the stabilization of benzoic acid within the 1HD2 enzyme. A closer examination of the docking poses of the most active synthesized compounds revealed the presence of hydrogen bonds with Cys47 and/or the key residues within the 1HD2 enzyme, confirming their antioxidant potential. The 3D and 2D representations gave a better comprehension of the ligand–protein interactions (Figure 4).

### 2.4. Molecular Dynamics Simulations

**Figure 5:**
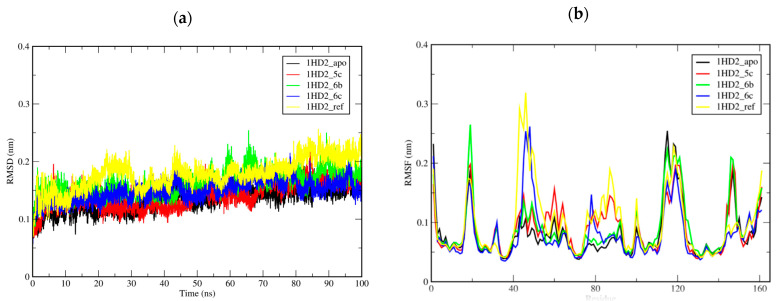
RMSD of c-α backbone (**a**) and RMSF for c-α atoms (**b**) of the 1HD2_apo, 1HD2_**5c**, 1HD2_**6b**, 1HD2_**6c**, and 1HD2_ref (ref = benzoic acid).

Molecular dynamics simulation (MDS) is a comprehensive analytical tool for evaluating the dynamic stability of a system. In this study, we conducted a 100 ns MDS of an unbounded 1HD2 and 1HD2 enzyme in a complex with the most active compounds (**5c**, **6b**, and **6c**) and with its co-crystalized ligand (benzoic acid). To study the stability and flexibility of these systems, two measures, root-mean-square deviation (RMSD) and root mean square flexibility (RMSF), were used to calculate the average movement of all atoms in the studied systems. The RMSD is a valuable metric for interpreting a system’s stability and conformational changes over simulation time. The RMSD plot (Figure 5a) shows that the RMSD averages over 100 ns for the systems of 1HD2 in complex with **5c** (1HD2_**5c**; 0.141 ± 0.024 nm), **6b** (1HD2_**6b**; 0.162 ± 0.022 nm), and **6c** (1HD2_**6c**; 0.149 ± 0.019 nm) were less than the average RMSD for 1HD2_ref (0.178 ± 0.028 nm) and were slightly larger than the RMSD of the apo form of 1HD2 (1HD2_ref; 0.135 ± 0.022 nm), indicating that they maintain stable structures. RMSF analysis provides information about the flexibility and dynamic behavior of individual residues of a system over the simulation time. The average RMSF value for 1HD2_**6c** (0.072 ± 0.047 nm) was lower than the average RMSF value for apo form of 1HD2 (0.074 ± 0.048 nm), while the averages for 1HD2_**5c** and 1HD2_**6b** were 0.080 ± 0.046 and 0.080 ± 0.050 nm, respectively, and 0.089 ± 0.060 nm for the reference ligand (Figure 5b). The systems exhibited low, stable fluctuation during the simulation, indicating the high rigidity of the systems. These two results confirmed that compounds **5c**, **6b**, and **6c** are tightly bonded and do not perturb the overall structural constancy of 1HD2 in addition to structural integrity.

### 2.5. Absorption, Distribution, Metabolism, Excretion and Toxicity (ADMET), and Pharmacokinetic Studies of the Synthesized Compounds

The six synthesized compounds’ physicochemical, ADMET, and drug-likeness characteristics were assessed using the SwissADME online software (http://www.swissadme.ch/) (Table 3). The outcomes presented in Table 3 show that most of the compounds complied with the rules of Lipinski and Veber. However, a violation was noted for compounds **5c** and **6c** due to their higher lipophilicity. None of the prepared derivatives exhibited Pan-assay interference compounds (PAINS) alerts. In addition, all derivatives displayed a high gastrointestinal absorption score (0.55), indicating promising oral bioavailability. No inhibitory interactions with CYP450-1A2 were observed, suggesting a favorable safety profile with minimal impact on drug metabolism. In terms of crossing the blood–brain barrier (BBB), all compounds were capable of doing so, except for **5b**.

None of the compounds exhibited practical resistance regarding P-glycoprotein (P-gp), contributing to multidrug resistance. Moreover, toxicity prediction was performed using the ProTox-II web tool (Table 3). The median lethal dose (LD_50_) value of **5b** (2450 mg/kg) falls into the fifth class, indicating a relatively low toxicity level. In comparison, other compounds are classified within the fourth category. Reference compounds such as ascorbic acid, BHT, and benzoic acid had LD_50_ values of 3367, 650, and 290 mg/kg, respectively, and were assigned to the fifth, fourth, and third classes. These findings suggest that the synthesized compounds exhibit toxicity profiles ranging from relatively low to moderate, with none classified as very toxic.

## 3. Materials and Methods

### 3.1. General Procedure

Melting points were uncorrected in an open capillary tube on a Buchi 510 apparatus. Spectra were recorded with the following instruments: ^1^H NMR (AC-300) and ^13^C NMR (AC-75) spectra were recorded on Bruker spectrometers (Singapore) with chemical shift values (d) given in part per million (ppm) relative to TMS (0.00 ppm). Mass spectra: Jeol JMS DX 300 (Tokyo, Japan). TMS was used as an internal reference. Column chromatography was carried out using E-Merck silica gel 60F254. The reaction progress was monitored by layer chromatography (TLC) using silica gel 60-F254, and the spots were detected with UV light (254 nm). The chemical structures of all the compounds were ascertained through analytical and spectroscopic methodologies, supplemented by comparing the obtained data with information available in the scientific literature regarding similar compounds. Following common laboratory practices, standard purification procedures were applied to the reagents and solvents.

### 3.2. Synthesis of Benzimidazolones ***2*** and ***3***

The benzimidazole derivatives **2** and **3** were prepared according to reported procedure [54] using 1-(cyclohex-1-enyl)-1,3-dihydro-2*H*-benzimidazol-2-one (**1**) (9.34 mmol), potassium carbonate (39.36 mmol), benzyltriethylammonium chloride (1.28 mmol) in acetone (30 mL) and agitated after 15 min. Next, the alkylating agent was added (15.77 mmol), and after stirring for 4–6 h at room temperature, the solvent was removed by evaporation. The organic layer was then subjected to extraction with dichloromethane, followed by drying using anhydrous sodium sulfate. Subsequently, the solvent was evaporated under vacuum. Compounds (**2**) or (**3**) were isolated by column chromatography, using hexane/ethyl acetate mixtures as an eluent.

### 3.3. General Procedure for Synthesis of Compounds ***5*** and ***6***

We successively introduced in a 100 mL flask equipped with a condenser and a CaCl_2_ guard, 10 mmol of *N*-propargylbenzimidazol-2-one (**2**) or *N*-allylbenzimidazol-2-one (**3**) and 9.10 mmol of hydrazonoyl bromide in 20 mL of anhydrous toluene. A total of 4 mL of triethylamine was carefully added to this mixture using a dropping funnel. The resulting mixture was then subjected to magnetic stirring and heated to reflux for 48–72 h. Triethylamine hydrochloride was formed during this reaction and filtered out while still hot. The solvent and excess triethylamine were subsequently removed using a rotary evaporator. The remaining residue was purified by column chromatography using hexane/ethyl acetate (8/2) as the eluent. The final product obtained from this process was further purified by recrystallization from ethanol to yield the desired compounds.

**Ethyl 5-[(3-(cyclohex-1-enyl)-2-oxo-2,3-dihydro-1*H*-benzimidazol-1-yl)methyl]-1-phenyl-1*H*-pyrazole-3-carboxylate: (5a)**

White Solid, Yield = 60%, M.p: 156–158 °C (ethanol). ^1^H NMR (300 MHz, CDCl_3_), δ (ppm): 1.30 (t, J = 7.07 Hz, 3H, CH_3_-CH_2_-O-), 1.66, 1.77, 2.23 (3m, 8H, 4CH_2_-cyclohexenyl), 4.33 (q, J = 7.11 Hz, 2H, CH_3_-CH_2_-O-), 5.04 (s, 2H, pyrazole-CH_2_-benzimidazolone), 5.81 (m, 1H, HC=C, C-cyclohexenyl), 6.49 (d, 1H, =HC, H-pyrazolic-4’), 6.77–7.39 (m, 9H, CH, H-Ar). ^13^C NMR (75 MHz, CDCl_3_), δ (ppm): 14.49 (1C, CH_3_-CH_2_-O-), 21.50, 22.15, 24.69, 26.73 (4C, 4 -CH_2_, C-cyclohexenyl), 36.80 (1C, pyrazole-CH_2_-benzimidazolone), 61.18 (1C, CH_3_-CH_2_-O-), 127.54 (1C, -HC=C, C-cyclohexenyl), 109.50 (1C, =CH, C-pyrazolic-4’), 107.78, 108.82, 121.49, 125.88, 129.37 (8C, =CH, C-Ar), 128.89, 129.54, 131.90, 139.54, 144.20 (6C, =C), 152.42, 162.21 (2C, C=O, CO_2_Et). HRMS of [M + H]^+^ m/z, calcd for C_26_H_27_N_4_O_3_: 443.20777, found: 443.20777.

**Ethyl 5-[(3-(cyclohex-1-enyl)-2-oxo-2,3-dihydro-1*H*-benzimidazol-1-yl)methyl]-1-p-tolyl-1*H*-pyrazole-3-carboxylate: (5b)**

White Solid, Yield = 68%, M.p: 164–166 °C (ethanol). ^1^H NMR (300 MHz, CDCl_3_), δ (ppm: 1.3 (t, *J* = 7.10 Hz, 3H, CH_3_-CH_2_-O-), 1.67, 1.77, 2,21 (3m, 8H, 4CH_2_-cyclohexenyl), 2.32 (s, 3H, p-CH_3_-Ar), 4.29 (q, *J* = 7.12 Hz, 2H, CH_3_-CH_2_-O-) 5.02 (s, 2H, pyrazole-CH_2_-benzimidazolone), 5.78 (m, 1H, HC=C, C-cyclohexenyl), 6.49 (d, 1H, =HC, H-pyrazolic-4’), 6.74–7.24 (m, 8H, CH, H-Ar). ^13^C NMR (75 MHz, CDCl_3_), δ (ppm): 14.36 (1C, CH_3_-CH_2_-O-), 21.19 (1C, p-CH_3_-Ar), 21.59, 22.54, 24.68, 26.72 (4C, 4 -CH_2_, C-cyclohexenyl), 36.78 (1C, pyrazole-CH_2_-benzimidazolone), 61.08 (1C, CH_3_-CH_2_-O-), 127.38 (1C, -HC=C, C-cyclohexenyl), 109.33 (1C, =CH, C-pyrazolic-4’), 107.82, 108.75, 121.44, 121.66, 125.53, 129.87 (6C, =CH, C-Ar), 128.49, 129.54, 132.02, 136.07, 139.40, 139.66, 144.09 (7C, =C), 152.42, 162.21 (2C, C=O, CO_2_Et). HRMS of [M + H]^+^ m/z, calcd for C_27_H_29_N_4_O_3_: 457.2234, found: 457.2234.

**Ethyl 1-(4-chlorophenyl)-5-[3-(cyclohex-1-enyl)-2-oxo-2,3-dihydro-1*H*-benzimidazol-1-yl)methyl]-1*H*-pyrazole-3-carboxylate: (5c)**

White Solid, Yield = 73%, M.p: 172–174 C (ethanol). ^1^H NMR (300 MHz, CDCl_3_), δ (ppm): 1.30 (t, *J* = 7.13 Hz, 3H, CH_3_-CH_2_-O-), 1.64, 1.76, 2.19 (3m, 8H, CH_2_-, H-cyclohexenyl), 4.32 (q, *J* = 7.12 Hz, 2H, CH_3_-CH_2_-O-), 5.05 (s, 2H, pyrazole-CH_2_-benzimidazolone), 5.76 (m, 1H, HC=C, C-cyclohexenyl), 6.52 (d, 1H, =HC, H-pyrazolic-4’), 6.82 -7.33 (m, 8H, CH, H-Ar). ^13^C NMR (75 MHz, CDCl_3_), δ (ppm): 14.35 (1C, CH_3_-CH_2_-O-), 21.56, 22.53, 24.69, 26.72 (4C, 4CH_2_-, C-cyclohexenyl), 36.73 (1C, pyrazole-CH_2_-benzimidazolone), 61.27 (1C, CH_3_-CH_2_-O-), 127.58 (1C, HC=C, C-cyclohexenyl), 109.88 (1C, =CH-, C-pyrazolic-4’), 107.75, 108.84, 121.54, 121.81, 126.89, 129.49 (=CH-Ar), 128.31, 129.42, 131.87, 135.31, 136.99, 139.61, 144.56, (7C, =C), 152.31, 161.99 (2C, C=O, CO_2_Et). HRMS of [M + H]^+^ m/z, calcd for C_26_H_26_ClN_4_O_3_: 477.1988, found: 477.1680.

**Ethyl 5-[(3-(cyclohex-1-enyl)-2-oxo-2,3-dihydro-1*H*-benzimidazol-1-yl)methyl]-1-phenyl-4,5-dihydro-1*H*-pyrazole-3-carboxylate: (6a)**

White Solid, Yield = 65%, M.p: 116–118 °C (ethanol). ^1^H NMR (300 MHz, CDCl_3_), δ (ppm): 1.41 (t, *J* = 7.06 Hz, 3H, CH_3_-CH_2_-O-), 1.76, 1.90, 2.31, 2.39 (4m, 8H, CH_2_-cyclohexenyl), 3.24 (s, 2H, pyrazole-CH_2_-benziùidazolone), 3.79, 4.20 (2m, 2H, -CH_2_, H-pyrazolic-4’), 4.36 (q, *J* = 7.04 Hz, 2H, CH_3_-CH_2_-O-), 5.15 (m, 1H, HC, H-pyrazolic-5’), 5.96 (m, 1H, HC=C, H-cyclohexenyl), 6.93–7.43 (m, 8H, H-Ar). ^13^C NMR (75 MHz, CDCl_3_), δ (ppm): 14.37 (1C, CH_3_-CH_2_-O-), 21.62, 22.57, 24.56, 26.95 (4C, 4CH_2_-, C-cyclohexenyl), 35.88, 41.67 (2C, 1C, C-CH_2_-, pyrazole-CH_2_-benzimidazolone, 1C, =CH-, C-pyrazolic-4’), 58.77 (1C, CH, C-pyrazolic-5’), 61.47 (1C, CH_3_- CH_2_-O-), 127.77 (1C, C=CH, C-cyclohexenyl), 107.92, 109.10, 114.56, 121.60, 129.40 (7C, CH, C-Ar), 129.84, 132.22, 138.98, 142.14 (6C, =C), 153.56, 162.63 (2C, C=O, C=O (CO_2_Et)). HRMS of [M + H]^+^ m/z, calcd for C_26_H_29_N_4_O_3_: 445.22342, found: 445.22342.

**Ethyl 5-[(3-(cyclohex-1-enyl)-2-oxo-2,3-dihydro-1*H*-benzimidazol-1-yl)methyl]-1-(p-tolyl)-4,5-dihydro-1*H*-pyrazole-3-carboxylate: (6b)**

White Solid, Yield = 70%, M.p = 140–142 °C (ethanol). ^1^H NMR (300 MHz, CDCl_3_), δ (ppm): 1.3 (t, *J =* 7.09 Hz*,* 3H, CH_3_-CH_2_-O-), 1.64, 1.76, 2.20, 2.24 (4m, 8H, CH_2_-cyclohexenyl and 3H, p-CH_3_-Ar), 3.13 (s, 2H, pyrazole-CH_2_-benzimidazolone), 3.49; 3.78 (2m, 2H, -CH_2_, H-pyrazolic-4’), 4.25 (m, 2H, CH3-CH_2_-O-), 5.31 (m, 1H, HC, H-pyrazolic-5’), 5.81 (m, 1H, HC=C, H-cyclohexenyl), 6.80–7.28 (m, 8H, H-Ar). ^13^C NMR (75 MHz, CDCl_3_), δ (ppm): 14.35 (1C, CH_3_-CH_2_-O-), 20.56 (1C, p-CH_3_-Ar), 21.62, 22.54, 24.69, 26.67 (4C, 4CH_2_-, C-cyclohexenyl), 36.02, 41.98 (2C, 1C, C-CH_2_-, pyrazole-CH_2_-benzimidazolone, 1C, =CH-, C-pyrazolic-4’), 61.80 (1C, CH, C-pyrazolic-5’), 61.25 (1C, CH_3_- CH_2_-O-), 127.32 (1C, C=CH, C-cyclohexenyl), 107.51, 108.63, 121.34, 121.49, 126.74, 128.99, 133.90 (7C, CH, C-Ar), 116.74, 129.48, 132.07, 137.52, 139.17, 141.52 (6C, =C), 153.12, 162.46 (2C, C=O, C=O (CO_2_Et)). HRMS of [M + H]^+^ m/z, calcd for C_27_H_31_N_4_O_3_: 459.23907, found: 459.23907.

**Ethyl 1-(4-chlorophenyl)-5-[(3-(cyclohex-1-enyl)-2-oxo-2,3-dihydro-1*H*-benzimidazol-1-yl)methyl]-4,5-dihydro-1*H*-pyrazole-3-carboxylate: (6c)**

White Solid, Yield = 75%, M.p = 128–130 °C (ethanol). ^1^H NMR (300 MHz, CDCl_3_), δ (ppm): 1.3 (t, *J* = 7.10 Hz, 3H, CH_3_-CH_2_-O), 1.66, 1.77, 2.21, 2.28 (4m, 8H, 4CH_2_-, H-cyclohexenyl), 3.12 (m, 2H, pyrazole-CH_2_-benzimidazolone), 3.65, 4.05 (2m, 2H, C-CH_2_-, H-pyrazolic-4’), 4.25 (q, *J* = 6.93 Hz, 2H, CH_3_-CH_2_-O), 5.01 (m, 1H, -CH, H-pyrazolic-5’), 5.85 (m, 1H, HC=C, C-cyclohexenyl), 6.79–7.27 (m, 8H, CH-Ar). ^13^C NMR (75 MHz, CDCl_3_), δ (ppm): 14.35 (1C, CH_3_-CH_2_-O), 21.61, 22.56, 24.73, 26.20 (4C, 4CH_2_-, C-cyclohexenyl), 36.20, 41.75 ((2C, 1C, pyrazole-CH_2_-benzimidazolone, 1C, C-CH_2_-, C-pyrazolic-4’), 58.72 (1C, CH, C-pyrazolic-5’), 61.35 (1C, CH_3_-CH_2_-O-), 127.72 (1C, C=CH cyclohexenyl), 107.16, 108.98, 115.73, 121.68, 129.26 (8C, CH-Ar), 126.52, 129.43, 129.55, 131.91, 139.63, 140.72 (6C, =C), 153.34, 162.42 (2C, C=O, C=O (CO_2_Et)). HRMS of [M + H]^+^ m/z, calcd for C_25_H_24_ClN_4_O_3_: 463.1531, found: 463.1531. HRMS of [M + H]^+^ m/z, calcd for C_26_H_28_ClN_4_O_3_: 479.18444, found: 479.18444.

### 3.4. Antioxidant Activity

The total antioxidant activity (TAC) and ferric reducing antioxidant power (FRAP) of the synthesized pyrazole derivatives were evaluated and measured.

#### 3.4.1. TAC by Phosphomolybdenum Method

The TAC of synthetized compounds was evaluated using Rafael Torres-Martínez et al. protocol [60]. A total of 100 μL of varying concentrations (14, 28, 56, 113, 226, and 452 μM) of the compounds in dimethylsulfoxide (DMSO) was combined with 900 μL of TAC reagent solution (0.6 M sulfuric acid, 28 mM sodium phosphate, and 4 mM ammonium molybdate). For the blank, 100 μL of distilled water was used in place of the compound. The tubes were incubated in a boiling water bath at 95 °C for 90 min. After the samples were cooled at room temperature, the absorbance of each sample was measured at 695 nm. The total antioxidant activity was calculated by the following equation: TAC (%) = ([*A*_sample_ − *A*_control_]/*A*_blank_) × 100.

#### 3.4.2. Ferric Reducing Antioxidant Power (FRAP)

According to the work of Nosipho Cele et al. [61], with a slight modification, 500 µL of varying concentrations (18, 35, 70, 141, 282, and 565 μM) of the compounds (500 µL) in DMSO was added to 100 µL of phosphate buffer (pH = 7.3) and 100 µL of 1% potassium ferricyanide [K_3_Fe(CN)_6_]. The mixture was incubated at 50 °C for 20 min, followed by acidification with 100 µL trichloroacetic acid TCA (10%). Then, 400 µL of the mixture was transferred into another test tube containing 300 µL of distilled water and 100 µL of FeCl_3_ (0.1%). Finally, the absorbance was read at 700 nm.

### 3.5. Computational Studies

#### 3.5.1. Molecular Docking

The remarkable antioxidant properties of compounds **5c**, **6b**, and **6c** prompted us to investigate their potential interactions with human peroxiredoxin 5 (PRDX5) as a target enzyme. The PRDX5 is crucial in antioxidant protection mechanisms and intracellular signal transduction. In this regard, the crystal structure of PRDX5 was downloaded from the Protein Data Bank (RSCB) using the PDB ID code 1HD2 [62]. Docking simulations were conducted using AutoDock Vina [63] to predict how compounds **5c**, **6b**, and **6c** might interact with the active site of PRDX5. Before initiating the docking procedure, the protein was prepared by removing water molecules and the co-crystallized ligand (benzoic acid), adding polar hydrogens and Gasteiger charges, and saving in pdbqt format with the assistance of AutodockTools [64]. For ligand preparation, the benzoic acid and the compounds (**5c**, **6b**, and **6c**) were saved in pdbqt format using the same software. Molecular docking simulations were performed under specific parameters: xyz coordinates of 7.02, 41.72, 34.40, and grid box dimensions of 21 Å^3^.

#### 3.5.2. Molecular Dynamics Simulations (MDs)

To further analyze the binding behavior of the compounds (**5c**, **6b** and **6c**), molecular dynamics simulations were performed using GROMACS 2021.3 software [65]. The apo-form of PRDX5 (1HD2_apo) and the systems of 1HD2_Ref (benzoic acid), 1HD2-**5c**, 1HD2-**6b**, and 1HD2-**6c** were subjected to 100 ns. Before initiating the MDS, the CHARMM-GUI web server [66] was used to generate the initial input parameters and implement the CHARMM36m force field [67]. Each complex was solvated in a rectangular grid box in TIP3P water, and counterions (Na^+^ and Cl^-^) were added via Monte Carlo ion displacement to maintain the desired salt concentration of 0.15 M. The energy minimization was carried out for each system using the steepest descent algorithm with a maximum of 50,000 steps and a maximum force of 10.0 KJ/mol. Simulation conditions were set to a temperature of 303.15 K and an atmospheric pressure of 1.01325 bar. An equilibration of each system was performed using canonical (NVT) and isothermal-isobaric (NPT) ensembles. A 100 ns MD simulation was then conducted. The structural stability of the designed molecules within the active site of PRDX5 was assessed based on the dynamics trajectory results, utilizing the RMSD and RMSF. The obtained data were visualized and analyzed using Xmgrace software (https://plasma-gate.weizmann.ac.il/Grace/, accessed on 17 November 2023).

#### 3.5.3. Absorption, Distribution, Metabolism, Excretion and Toxicity (ADMET) Profile Estimation

Since pharmacokinetics, drug-likeness, and toxicity play a crucial role in drug discovery, conducting an ADMET profile of the synthesized compounds was essential. For this purpose, the predictions for these compounds were performed using the SwissADME and ProTox-II web tools [68,69].

#### 3.5.4. Statistical Analyses

The biological activities were determined in triplicate for each sample. The results were provided as means of the standard deviation (±SD).

## 4. Conclusions

This current study explored the interaction between benzimidazolone and various nitrilimines to synthesize heterocyclic compounds. These compounds were determined by spectroscopic analyses, including NMR (^1^H and ^13^C) and HRMS. The potential antioxidative efficacy of the synthesized compounds was analyzed by examining their capacity to neutralize radicals using FRAP and TAC methods. Compounds **5c**, **6b**, and **6c** exhibited the most significant antioxidant activity, suggesting their promising potential as antioxidants compared to ascorbic acid and BHT. It has been shown that the presence of methyl or chloride atoms in the phenyl group within the pyrazole ring significantly improves the antioxidant activity. In addition, the molecular docking and molecular dynamic simulation results confirmed their strong binding profile and the stability of their interactions within the active site of the PRDX5 enzyme. The ADMET study predicted the synthesized compounds’ good physicochemical and pharmacokinetic profiles. These findings revealed the potential antioxidant activity of these compounds and suggested that they could serve as a promising basis for further improvements in their structure to develop more potent antioxidant agents.

## Figures and Tables

**Table 1:** Antioxidant activity results of compounds **5**(**a**–**c**) and **6**(**a**–**c**).

Compound	IC_50_ (µM)	
	TAC	FRAP
**5a**	44.62 ± 0.20	108.30 ± 0.59
**5b**	247.80 ± 1.54	197.93 ± 2.05
**5c**	14.00 ± 0.14	120.50 ± 1.38
**6a**	56.55 ± 0.10	92.70 ± 0.43
**6b**	12.47 ± 0.02	388.50 ± 1.59
**6c**	12.82 ± 0.10	68.97 ± 0.26
Ascorbic Acid	65.97 ± 1.53	88.12 ± 0.23
BHT	31.76 ± 1.22	--

*p*-value ≤ 0.05.

**Table 2:** The docking summary of the most active compounds and the reference compounds and their binding interactions with the active site of PRDX5 (PDB ID: 1HD2).

	Binding Affinity (Kcal/mol)	Hydrogen Binding		Hydrophobic Interaction	
		NB	Residues	NB	Residues
**5c**	−6.2	0	-	7	Pro40, Pro45, Phe120
**6b**	−6.3	4	Thr44, Gly46, Cys47	4	Pro40, Pro45, Leu116, Phe120
**6c**	−6.3	4	Thr44, Gly46, Cys47	4	Pro40, Pro45, Leu116, Phe120
Ascorbic acid	−4.5	5	Thr44, Cys47, Arg127, Thr147	0	-
BHT	−4.6	0	-	6	Pro40, Pro45, Ile119, Phe120
Benzoic acid	−4.5	4	Thr44, Cys47, Arg127, Thr147	2	Pro40, Arg127

**Table 3:** The physicochemical, pharmacokinetic, and ADMET properties of synthesized compounds.

	5a	5b	5c	6a	6b	6c
Molecular weight (≤500 g/mol)		456.54	476.95	444.53	458.55	478.97
H-bond acceptors (≤10)	4	4	4	4	4	4
H-bond donors (≤5)	0	0	0	0	0	0
Rotatable bonds (≤10)	7	7	7	7	7	7
TPSA (≤140 Å^2^)	71.05	71.05	71.05	68.83	68.83	68.83
MLog *P*_o/w_ (≤4.15)	3.93	4.13	4.40	3.77	3.97	4.24
GI absorption	High	High	High	High	High	High
BBB permeant	Yes	No	No	Yes	Yes	Yes
P-gp substrate	No	No	No	No	No	No
CYP1A2 inhibitor	No	No	No	No	No	No
Lipinski’s rule (violation)	Yes (0)	Yes (0)	Yes (1)	Yes (0)	Yes (0)	Yes (1)
Veber’s rule	Yes	Yes	Yes	Yes	Yes	Yes
Bioavailability Score	0.55	Yes	Yes	Yes	Yes	Yes
Brenk	0	0	0	0	0	0
PAINS	0	0	0	0	0	0
LD_50_ (mg/kg body weight)	540	2450	564	1250	1350	1350
Predicted toxicity class	4	5	4	4	4	4

Abbreviations: BBB = blood–brain barrier; GI absorption = gastrointestinal absorption; LD_50_ = median lethal dose; MLog Po/w = Moriguchi n-octanol–water partition coefficient; PAINS = Pan-assay interference compounds; P-gp substrate = P-glycoprotein substrate; TPSA = Topological Polar Surface Area.

## Data Availability

Data are contained within the article and Supplementary Materials.

## Supplementary Materials

The following supporting information can be downloaded at https://www.mdpi.com/article/10.3390/ph16121648/s1. ^1^H-NMR, ^13^C-NMR and HRMS spectrums of synthesized compounds.
