# Supplementary material for: Design, Synthesis, Molecular Modeling and Biological Evaluation of Novel Pyrazole Benzimidazolone Derivatives as Potent Antioxidants
Source: Pharmaceuticals (Basel). 2023 Nov 24;16(12):1648. doi: 10.3390/ph16121648 (PMC10747449; doi:10.3390/ph16121648)

Supplementary Information for

Design, Synthesis, Molecular Modelling and Biological Evaluation of Novel Pyrazole Benzimidazolone Derivatives as Potent Antioxidants

Mohamed Adardour,[1,*] Marouane Ait Lahcen,[1] Mehdi Oubahmane,[1] Walid Ettahiri,[1,2] Ismail Hdoufane,[1] Hafida Bouamama, [3] Mohammed M. Alanazi,[4] Driss Cherqaoui,[1,5] Mustapha Taleb,[2] Elena Zaballos Garcia,[6] and Abdesselam Baouid [1]

[1] Molecular Chemistry Laboratory, Department of Chemistry, Semlalia Faculty of Sciences, 2390, Cadi Ayyad University, Marrakech 40001, Morocco; m.aitlahcen.ced@uca.ac.ma (M.A.L.); mehdi.oubahmane@ced.uca.ma (M.O.); walid.ettahiri@usmba.ac.ma (W.E.); i.hdoufane@uca.ac.ma (I.H.); cherqaoui@uca.ac.ma (D.C.); baouid@uca.ac.ma (A.B.)

[2] Laboratory of Engineering, Electrochemistry, Modeling and Environment, Faculty of Sciences, Sidi Mohamed Ben Abdellah University, Fez 30000, Morocco; mustaphataleb62@yahoo.fr

[3] Laboratory of Sustainable Development and Health Research, Faculty of Sciences and Techniques, Cadi Ayyad University, Marrakech 40000, Morocco; h.bouamama@uca.ac.ma

[4] Department of Pharmaceutical Chemistry, College of Pharmacy, King Saud University, P.O. Box 2457, Riyadh 11451, Saudi Arabia; mmalanazi@ksu.edu.sa

[5] Sustainable Materials Research Center (SUSMAT-RC), University of Mohammed VI Polytechnic, Benguerir 43150, Morocco

[6] Department of Organic Chemistry, Faculty of Pharmacy, University of Valencia, Ave. Vte. Andres Estelles s/n, 46100 Valencia, Spain; elena.zaballos@uv.es

* Correspondence: mohamed.adardour@ced.uca.ac.ma

NMR and HRMS spectra for synthesis pyrazole

^{1}H NMR of Compound 5a

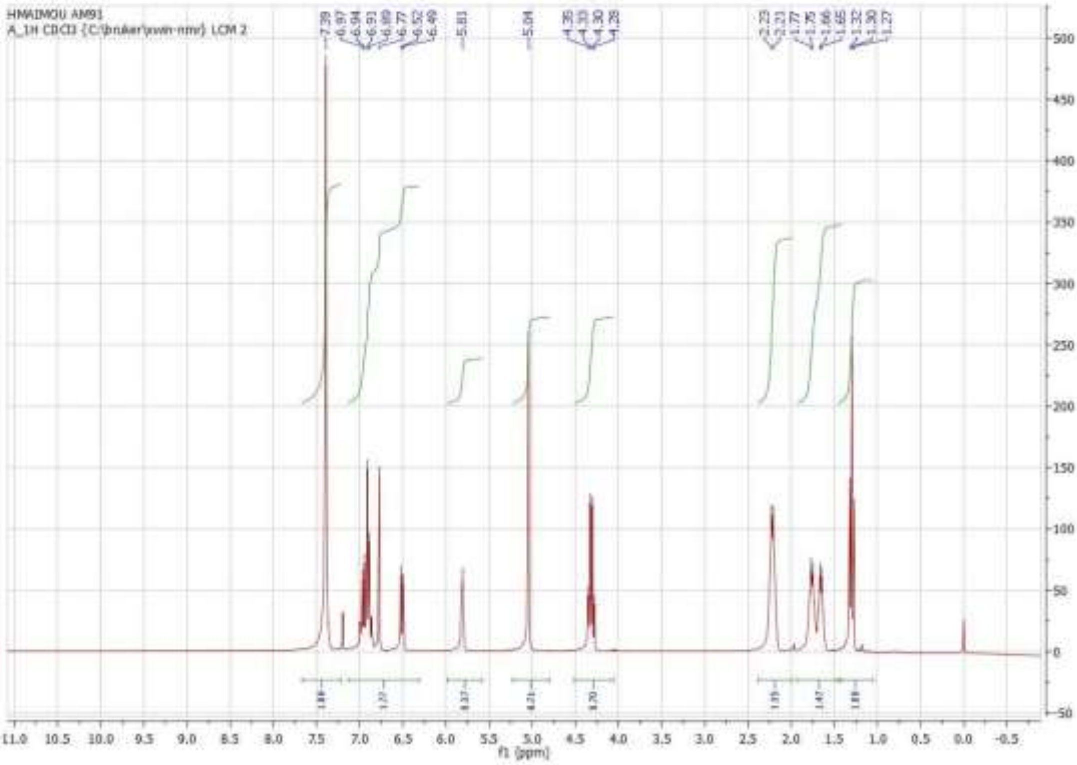

^{13}C NMR of Compound 5a

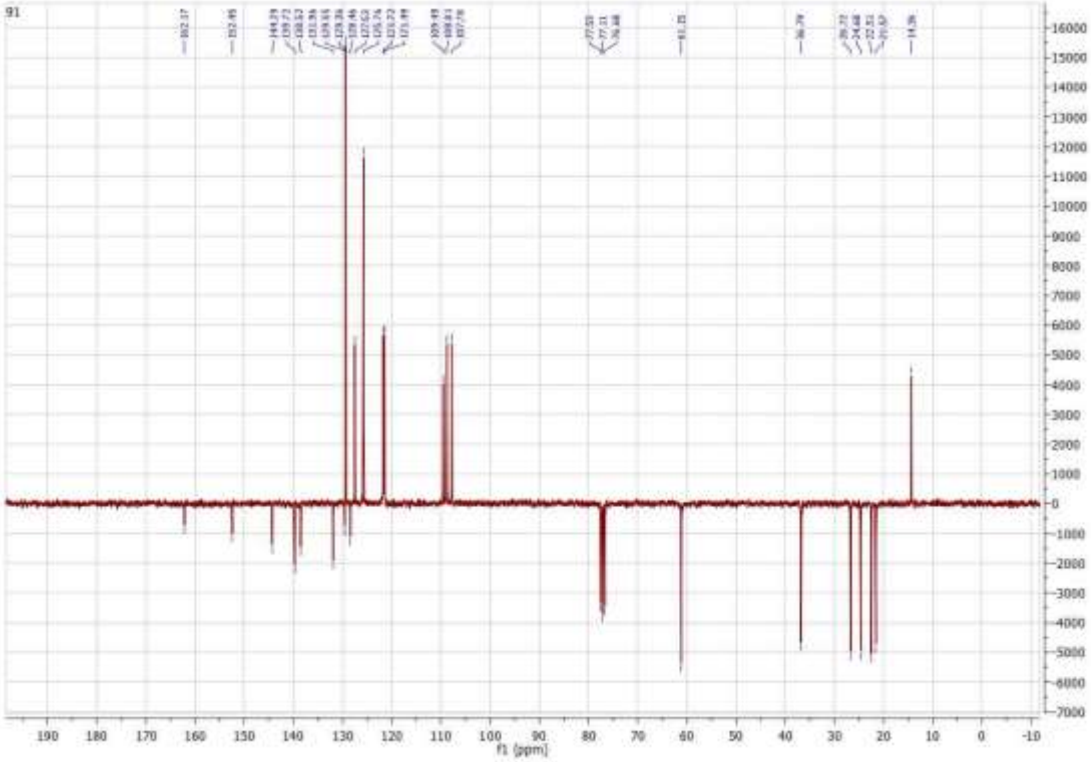

^{1}H NMR of Compound 5b

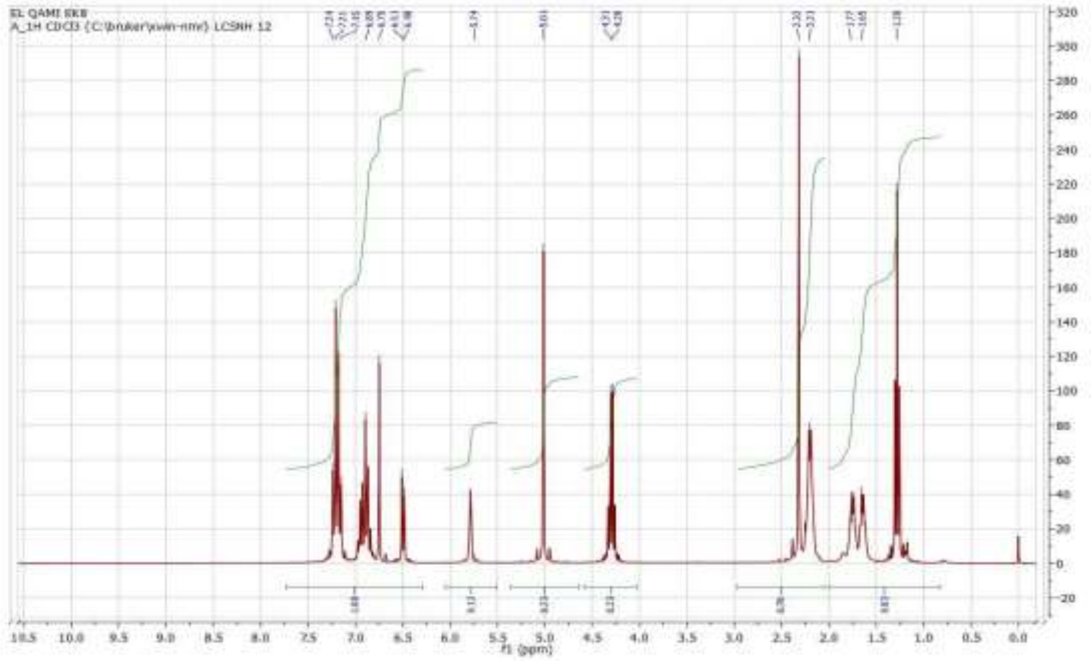

^{13}C NMR of Compound 5b

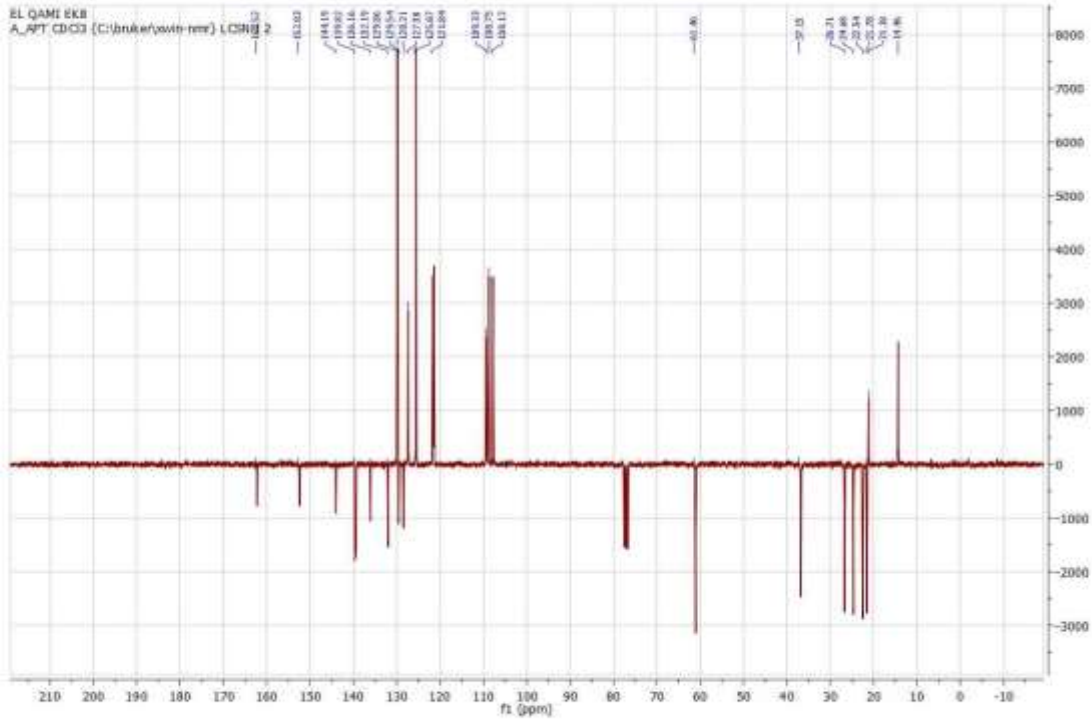

HRMS of Compound 5b

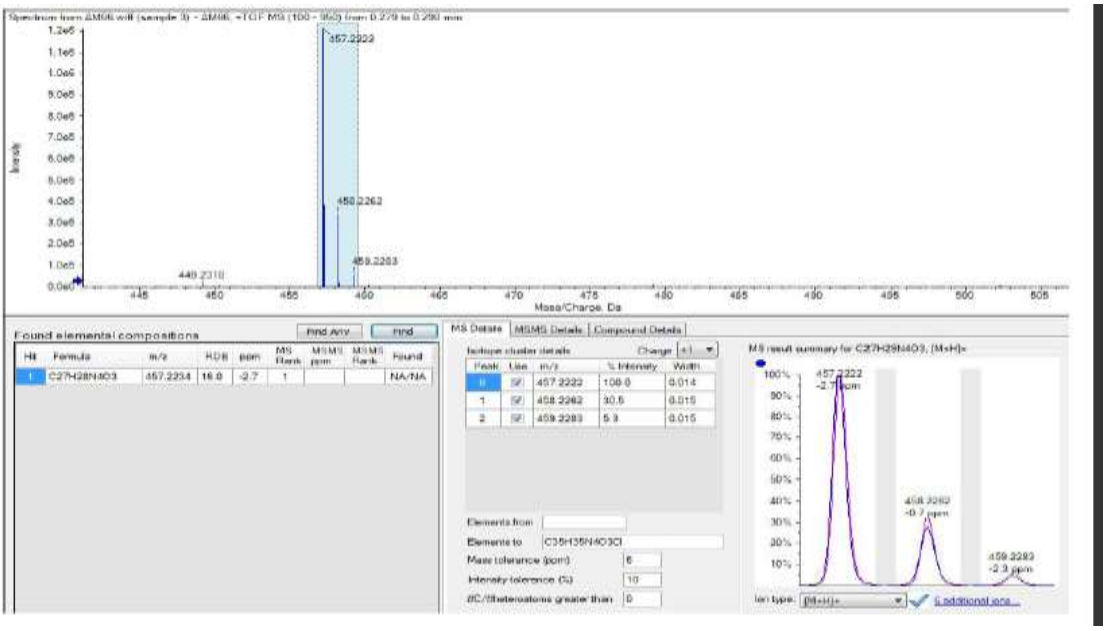

^{1}H NMR of Compound 5c

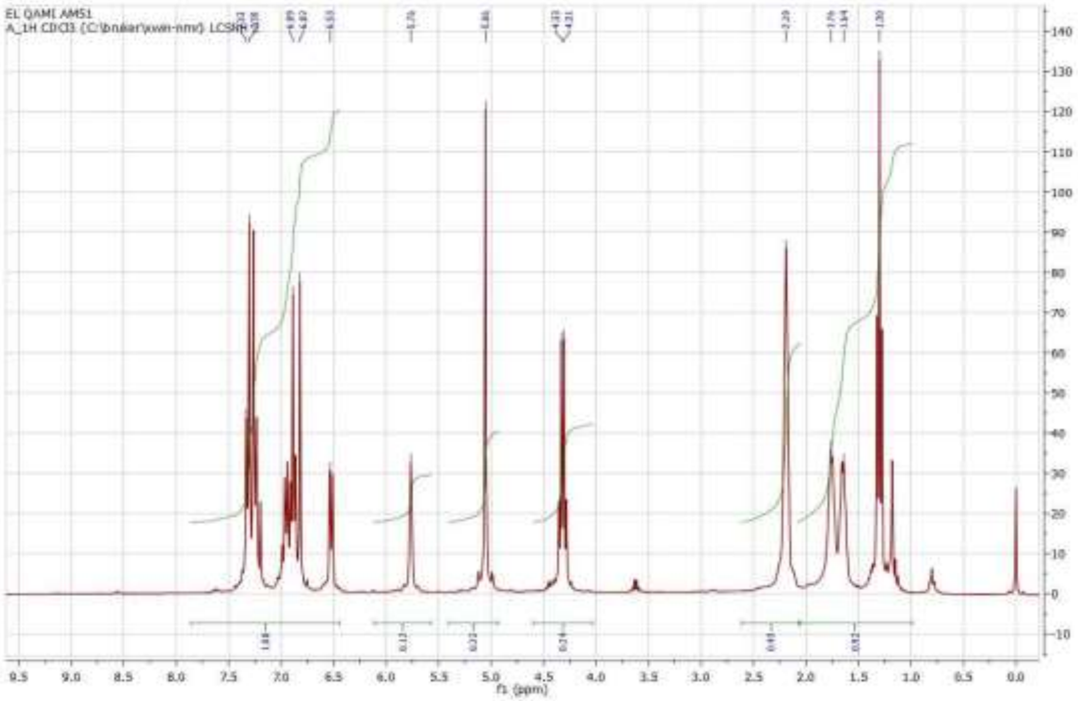

^{13}C NMR of Compound 5c

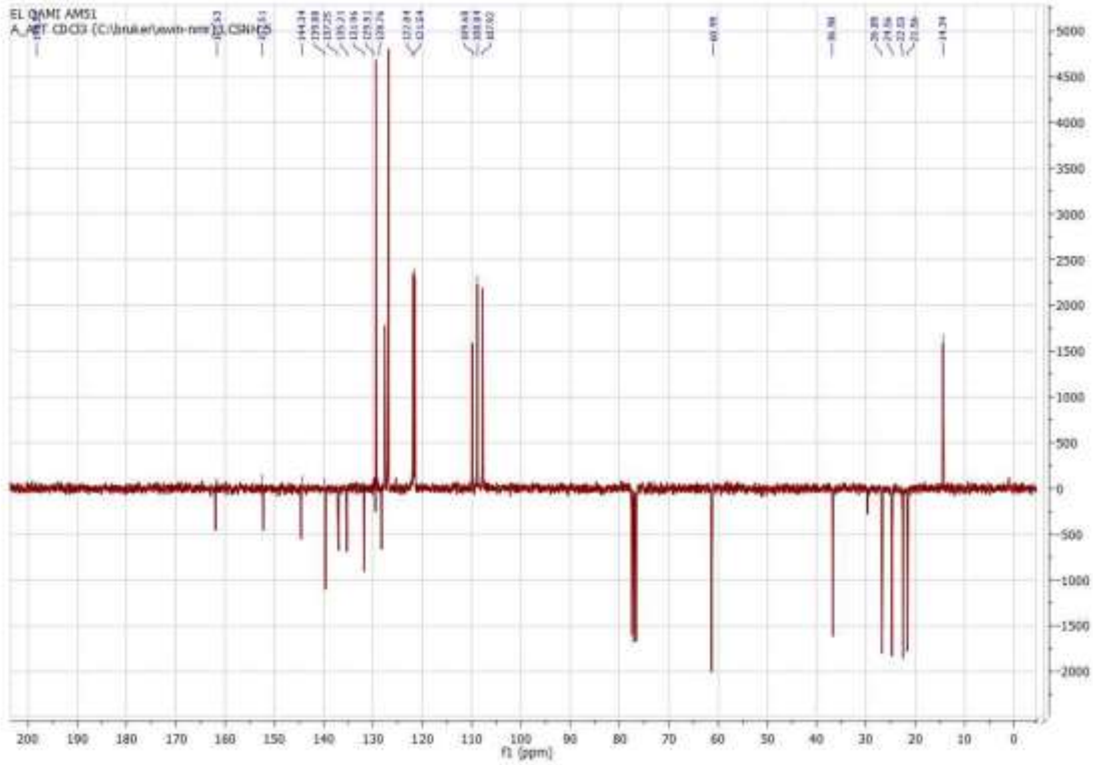

HRMS of Compound 5c

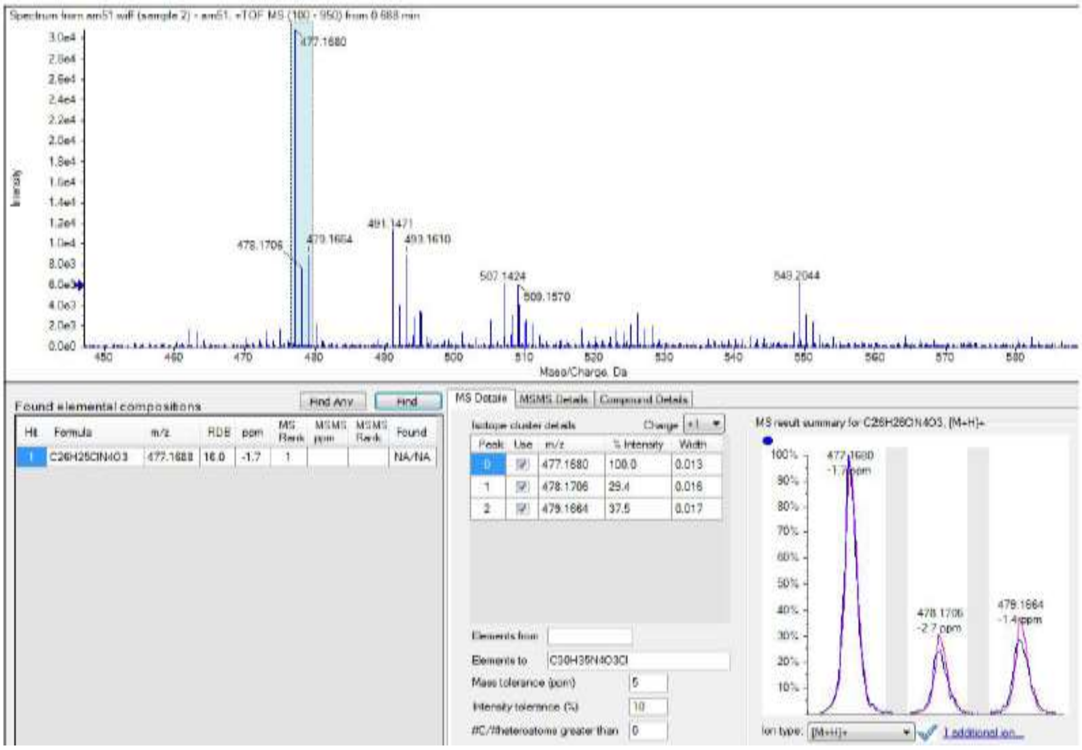

^{1}H NMR of Compound 6a

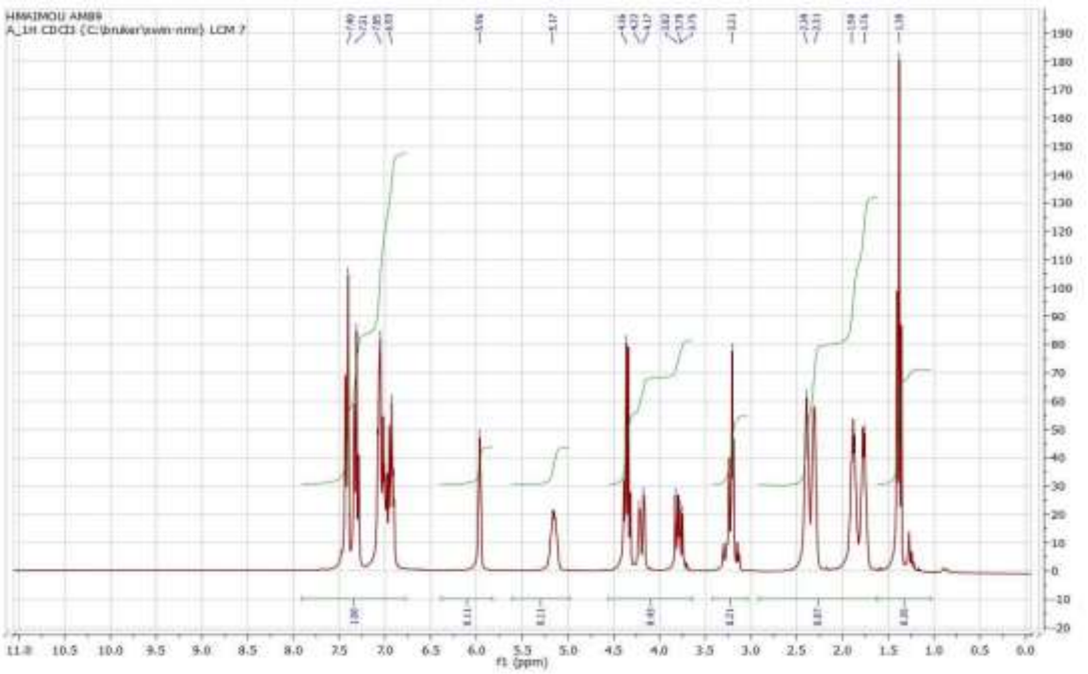

^{13}C NMR of Compound 6a

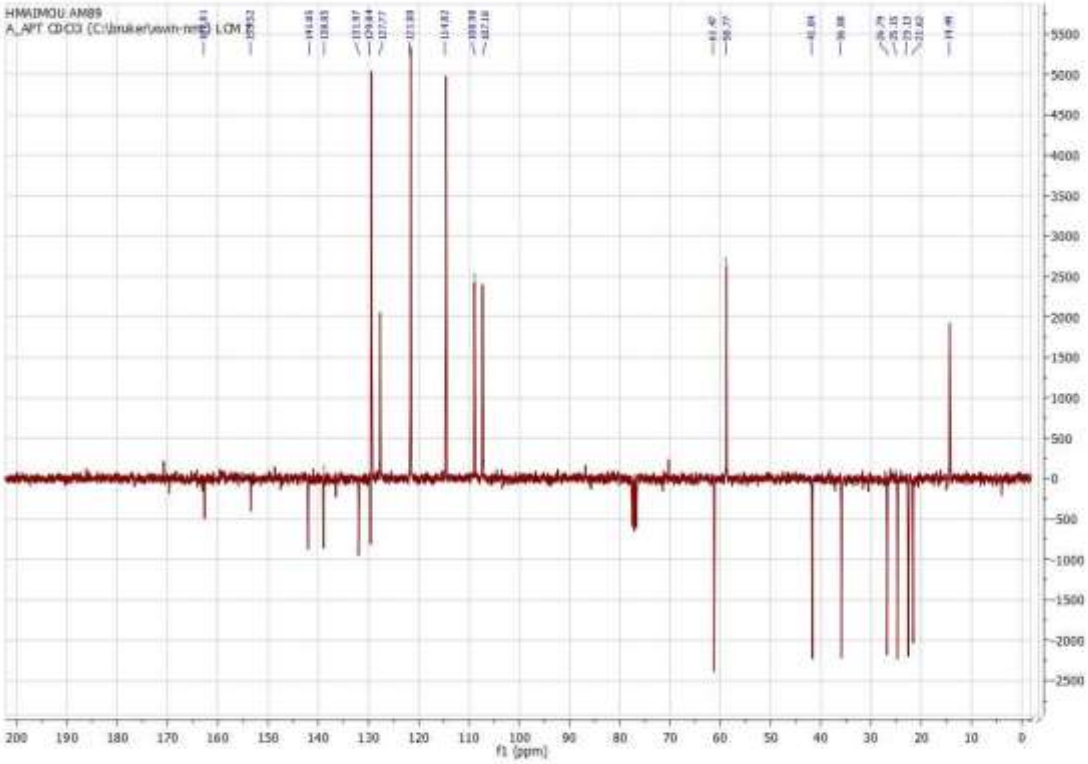

^{1}H NMR of Compound 6b

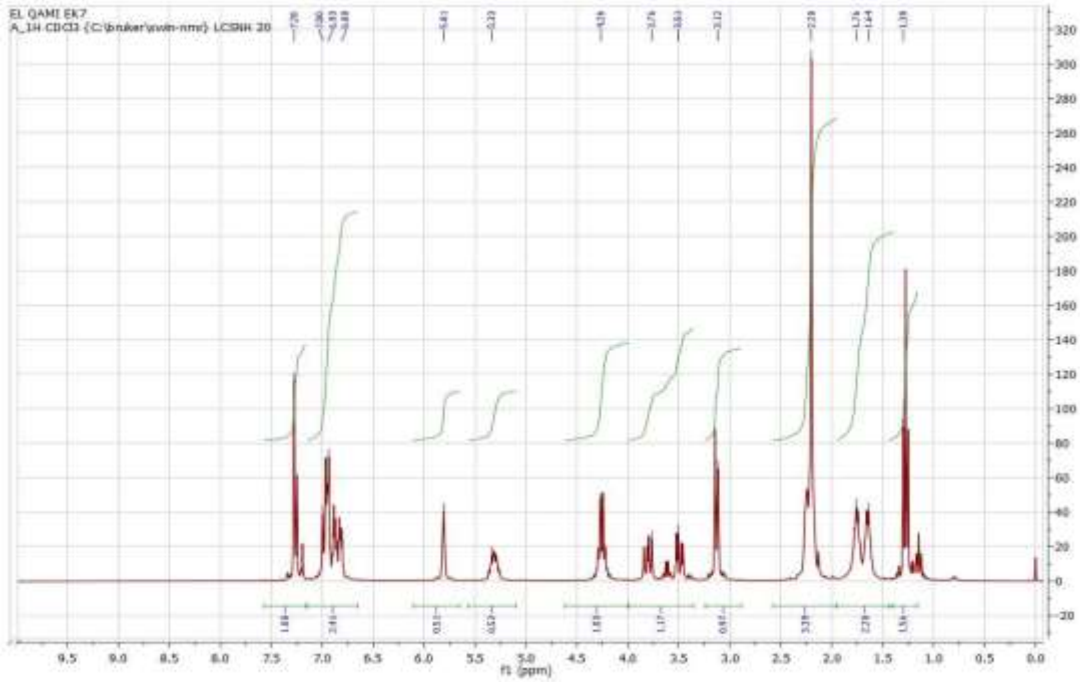

^{13}C NMR of Compound 6b

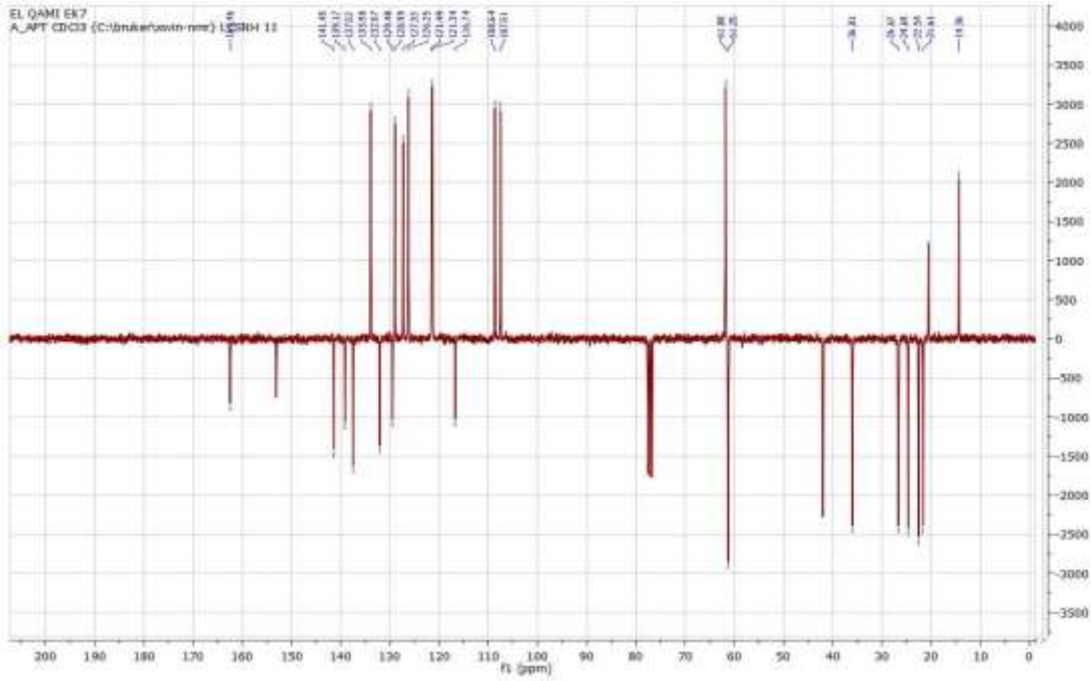

^{1}H NMR of Compound 6c

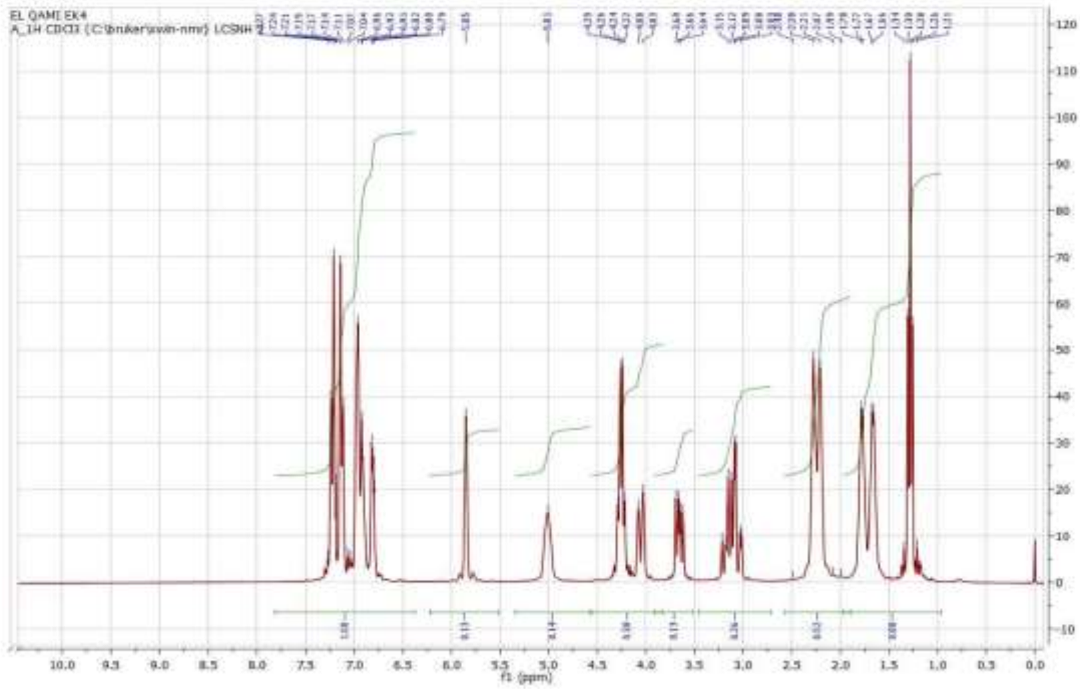

^{13}C NMR of Compound 6c

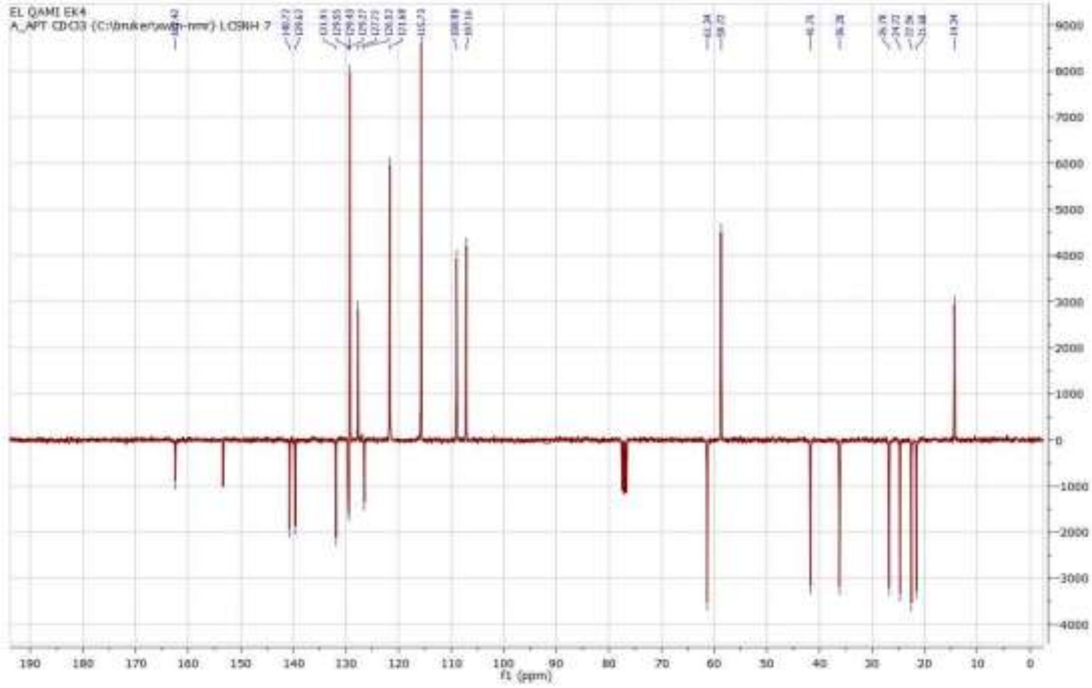

Supplement: Supplementary file 1 [file pharmaceuticals-16-01648-s001.zip › pharmaceuticals-2715283-supplementary.pdf]
